# Alveolar soft part sarcoma: the new primary intracranial malignancy: A case report and review of the literature

**DOI:** 10.1007/s10143-017-0874-4

**Published:** 2017-07-26

**Authors:** Aditaya Kumar, B. Alrohmain, W. Taylor, P. Bhattathiri

**Affiliations:** 1 Department of Neurosurgery, The Institute of Neurological Sciences, Glasgow, UK; 2 Flat 1/1, 7 Dryburgh Gardens, Glasgow, G20 6BT Glasgow UK

**Keywords:** Alveolar soft part sarcoma, Primary, Intracranial, Tumour

## Abstract

The purpose of this paper is to serve as a reference to aid in the management of this poorly understood intracranial malignancy. The authors report their experience treating the eighth ostensible case of a primary intracranial alveolar soft part sarcoma (ASPS). A 21-year-old man presented to hospital after collapsing. He gave a 1-year history of headache, a 2-month history of reduced visual acuity and on examination had left facial paraesthesia with left-sided incoordination. MRI of the brain revealed a large left posterior fossa mass. The patient underwent resection of the tumour with good recovery in function. Immunohistochemical analysis of the tumour specimen confirmed an ASPS, and multimodal imaging in search of an extra-cranial disease primary was negative. A review of the literature yielded only seven other cases of primary intracranial ASPS. A variety of diagnostic imaging modalities were employed in search of a disease primary, as were various combinations of surgical resection, chemotherapy and radiotherapy as treatment. Half of the cases documented delayed disease recurrence. The authors discuss the following: the unique radiological and immunohistological characteristics of this disease including the potential for its misdiagnosis; the investigations required to diagnose a primary intracranial ASPS; the efficacy of current medical and surgical treatment options and the factors that will aid in prognostication. This is the first review of this new primary intracranial malignancy. From our analysis, we offer a joint radiological and immunohistochemical algorithm for the diagnosis of primary intracranial ASPS and specific operative considerations prior to resection.

## Introduction

Alveolar soft part sarcoma (ASPS) is an extremely rare malignancy of unknown cellular origin accounting for 1% of all soft tissue sarcomas. ASPS effects a younger patient population with a peak incidence between 15 and 35 years [9]. The primary site for ASPS is most commonly the trunk or extremities, but it can also arise from the abdomen or the head and neck. Beyond its rarity, ASPS is also unusual in its characteristics of metastasis. Other high-grade soft tissue sarcomas rarely metastasize to the brain, whereas ASPS has a high metastatic potential and will involve the brain in up to 19% of cases [19]. To date, there are only ten cases of ASPS presenting with neurological symptoms, four of which had concomitant lung metastasis [4].

Here, we present the eighth ostensible case of primary intracranial ASPS and the second case of ASPS arising in the posterior fossa [1]. In conjunction with a review of the literature, we discuss the diagnosis, evidence for medical and surgical management, factors aiding in prognostication and surgical considerations for this new primary intracranial malignancy. Our aim is to provide a comprehensive reference for any clinicians who encounter this incompletely understood disease entity in the future.

## Case

### History and examination

A 21-year-old man presented to his local hospital after collapsing at home due to leg weakness. He gave a 1-year history of persistent headache accompanied by a 2-month history of blurred vision, reduced sensation on the left side of his face and occasional difficulty in walking. He had a history of learning difficulties and anxiety with no other medical problems. On admission, he was fully conscious with a Glasgow Coma Score of 15/15. Cranial nerve examination revealed a left 4th and 6th nerve palsy causing diplopia and reduced sensation in all distributions of the left trigeminal nerve. There was left-sided nystagmus in conjunction with left cerebellar signs causing a broad-based gait. Fundoscopy revealed papilloedema. Peripheral neurological examination was unremarkable.

### Investigations

**Fig. 1:**
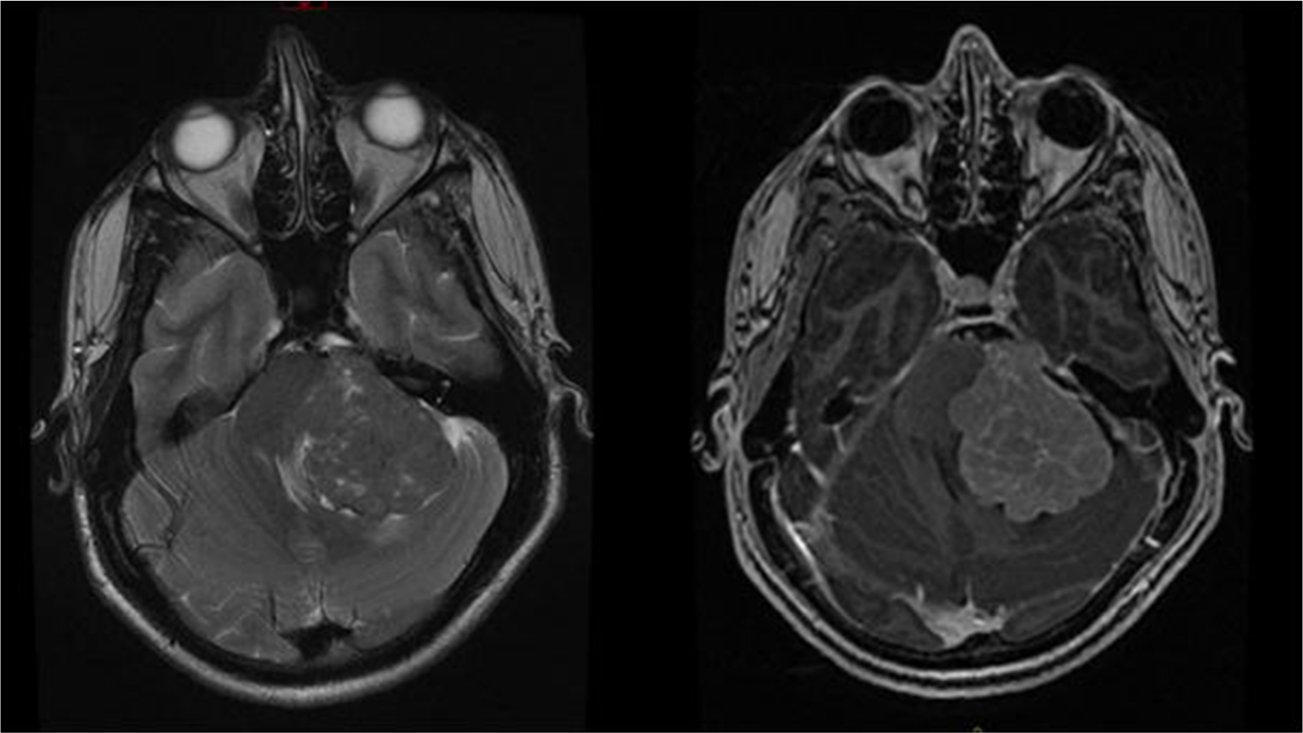
Pre-operative axial T2 pre-contrast (*left*) and T1 post-contrast (*right*) showing a large broad-based extra-axial mass measuring 4 × 4 × 5 cm in the left posterior fossa effacing and displacing the fourth ventricle

MRI brain with contrast revealed a broad-based and extra-axial mass measuring 4 × 4 × 5 cm in the left posterior fossa. It was isointense to grey matter on T1-weighted imaging (WI) and heterogeneous on T2WI imaging with avid heterogeneous T1WI enhancement post-gadolinium administration. Diffusion-weighted imaging showed no restriction with some small cystic components. Evidence of mass effect was noted on the left cerebellar lobe and the midbrain with evidence of hydrocephalus. Multiple small vessels were intimately associated with the lesion. An initial radiological diagnosis of meningioma was made (Fig. 1).

Audiometry showed a left sensironeural hearing loss >70 db in the left ear, and ophthalmology review confirmed bilateral papilloedema with 6/9 acuity in the right eye and 6/18 acuity in the left eye.

### Operation

The patient was positioned left side up in the park bench position with the head pinned. A left occipital external ventricular drain was placed to release cerebrospinal fluid under high pressure. Following this, a left suboccipital and retromastoid craniotomy was performed exposing the margins of the transverse and sigmoid sinuses. The dura was noted to be full after the bone flap was removed, and the cerebellum was tense and bulging after dural opening. Extra-axial tumour was encountered at 2 cm depth. The tumour was encapsulated but with no clear plane for dissection from the cerebellum and highly vascular. No definite site of attachment to the dura or brain was found. Microsurgical dissection was performed with neuro-physiological monitoring ensuring preservation of the 5th, 7th and lower cranial nerves. During dissection, severe bleeding was encountered, especially venous, which necessitated 13 units of packed red cell transfusion. The haemorrhage was only fully controlled after total microsurgical resection. The dura was then closed with bone flap replacement, and the patient transferred to intensive care.

### Pathology

Macroscopic analysis showed a mixed cream and brown rubbery tissue. Microscopic appearances showed distinct nests of medium to large plump epithelioid cells with prominent nuclei and a granular eosinophilic cytoplasm. The nests were intersected by numerous fine vessels and showed no evidence of necrosis. Staining revealed moderate numbers of cells with granular to needle-shaped cytoplasmic inclusions that were PAS positive and diastase-resistant. Immunocytochemistry was diffusely positive for myo-D1 with nuclear positivity for INI1. Stains for desmin, CK-MNF, Cam 5.2, chromogranin, synaptophysin, s100, EMA, smooth muscle actin, CD117, PLAP, CD30, GFAP, beta-hCG, AFP, hepar-1 and RCC were all negative. Given that a broad differential had been excluded, transcription factor E3 (TFE3) staining was performed and showed strong nuclear positivity confirming an alveolar soft part sarcoma.

### Post-operative course

Immediately post-operation, the patient had worsening of his cranial nerve dysfunction with a new left facial weakness. During the next few weeks, his cranial nerve palsies gradually improved, and he was eventually able to ambulate independently. At this point, he was referred to the oncology team for further investigation.

**Fig. 2:**
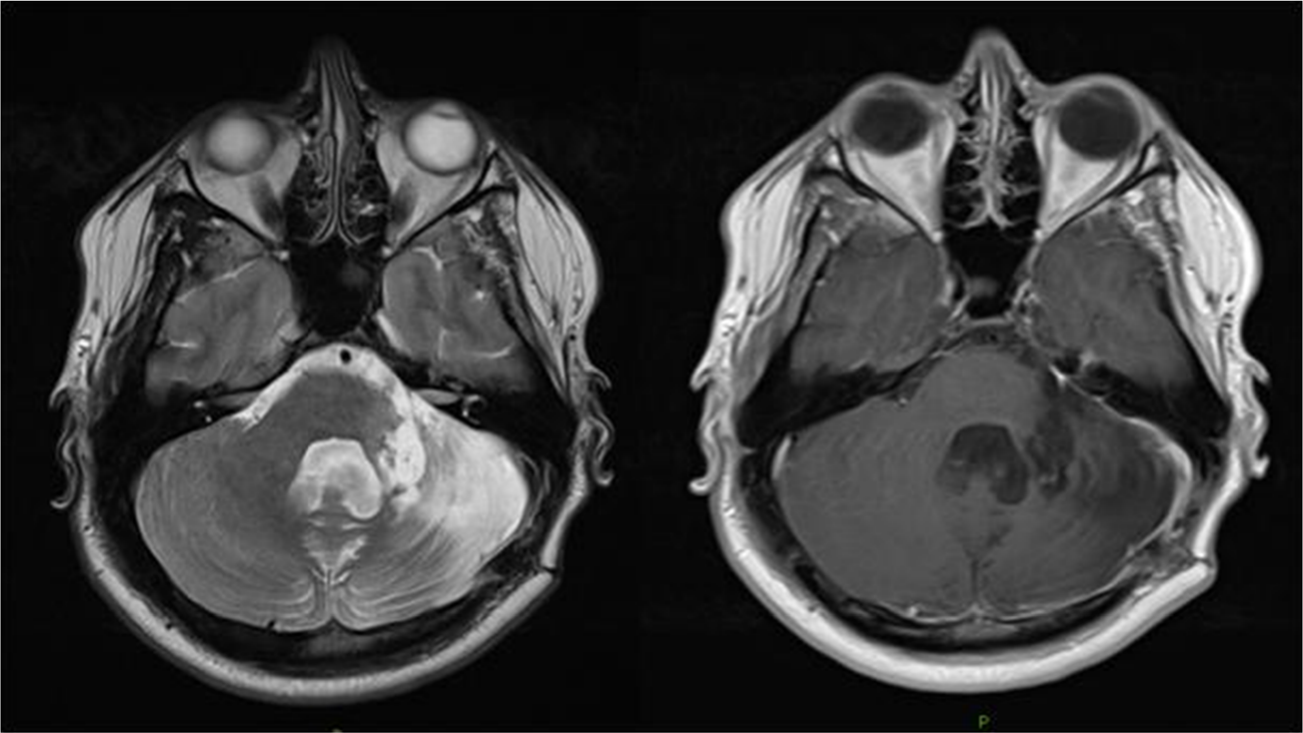
Post-operative axial T2 (left) and T1 MRI (right) brain post-contrast at 9 months post-resection. Evidence of associated gliosis with some haemosiderin deposit in the left cerebellar hemisphere. No pathological enhancement is seen in the area of the resection cavity to indicate tumour recurrence

Extensive imaging in search of a primary lesion site was all negative, including repeat whole-body FDG-PET scans over a 2-month period. At 10-month follow-up, he is living at home requiring no formal package of care. He is currently receiving regular follow-up as part of surveillance by his oncology team. A repeat MRI brain at this time showed no evidence of disease recurrence, and CT of the chest, abdomen and pelvis showed no disease primary (Fig. 2).

## Primary intracranial alveolar soft part sarcoma

It is known that ASPS has an unusual predilection for intracranial metastasis in comparison to other soft tissue sarcomas. It is reported that intracranial metastatic ASPS is only observed with concomitant extra-cranial metastatic disease and never in the absence of lung metastases [11, 20]. Given it is estimated that the accuracy of FDG-PET in unknown primary tumour detection has a sensitivity of 87% and specificity of 71% [8] and our patient exhibited no radiological evidence of metastatic lung disease, we posit that this is the eighth ostensible case of primary intracranial ASPS reported in the English literature.

An online search of the English literature for the terms “alveolar soft part sarcoma” and “primary” yielded 126 results. One hundred twenty-three of these were discarded as they did not pertain to intracranial disease. Review of references for the remaining three cases provided four further relevant cases giving a total of seven reports of primary intracranial ASPS [13] [22] (Table 1).

**Table 1:** Cases of reported primary intracranial ASPS to date

Case	Authors	Age, gender	Presentation	Location	Investigations to find primary	Treatment	Outcome
1	Bodi et al.	39, M	Seizures	Left temporal meningeal	CT chest/abdo/pelvis	Total surgical resection	Alive at 10 months
2	Das et al.	17, F	Frontal mass	Bifrontal	Abdo USS, CXR, bone scan	Total surgical resection, adjuvant chemotherapy	Alive at 4 months
3	Ahn et al.	9, F	Headache, seizure, tinnitus	Right cerebellopontine angle	Whole body PET, CT chest/abdo/pelvis, bone marrow biopsy	Subtotal surgical resection, radiotherapy, chemotherapy, ×2 radiosurgery for recurrence	New lesion at 14 months, local recurrence at 29 months
4	Emmez et al.	11, F	Headache, seizure, paraesthesia	Left frontal	CT chest/abdo/pelvis, CT sinuses, bone marrow biopsy, whole-body PET	Total surgical resection, whole brain radiotherapy, adjuvant chemotherapy, reoperation, 2nd course chemotherapy	Local recurrence at 45 months
5	Mandal et al.	32, F	Headache, vomiting, diplopia	Right parietal meningeal	Unknown	Total surgical resection	Unknown
6	Tao et al.	28, F	Forehead mass	Left frontal	CT chest, USS abdomen, whole-body PET	Total surgical resection, adjuvant radiotherapy	Alive at 27 months
7	Tao et al.	13, M	Tinnitus, proptosis	Right temporal	CT chest, USS abdomen	Subtotal surgical resection, adjuvant radiotherapy	Local recurrence, died at 24 months
8	Current case	21, M	Headache, ataxia, cranial nerve palsies	Left posterior fossa/anterior brainstem	CT chest, USS thorax, ×2 whole-body PET	Total surgical resection	Alive at 8 months

*CT* computerized tomography, *CTX* chemotherapy, *CXR* chest X-ray, *PET* positron emission tomography, *RTX* radiotherapy, *STR* subtotal surgical resection, *TSR* total surgical resection, *USS* ultrasound scan

In the eight cases of primary intracranial ASPS, a younger patient population is effected as would be expected. Furthermore, the age-related gender ratio inversion described by Ordonez is preserved in primary intracranial ASPS; the youngest cases being female and the older cases being male [17]. Presentation is usually due to symptoms of raised intracranial pressure or seizure with two cases presenting as a large frontal forehead mass.

## Diagnosis

An initial radiological diagnosis of meningioma was made in our case similarly to four of the other cases of primary intracranial ASPS. Typically, meningiomas are isointense to hypointense in comparison to grey matter on T1WI MRI with variable signal intensity on T2WI [19]. ASPS is most commonly known to be isointense to hyperintense on T1WI and characteristically hyperintense on T2WI, but this is in comparison to muscle [10].

As of the time of writing, there is no literature specifically addressing the MRI characteristics of ASPS with regards to T1 and T2WI when compared to grey matter. This is presumably because of the extreme rarity of the disease arising as a primary intracranial tumour and therefore the radiological diagnosis being made upon detection of the soft tissue primary and its characteristics relative to muscle.

Retrospective review of the MRI characteristics of our patient showed that the tumour satisfied criteria for a benign posterior fossa tumour, such as a meningioma, but also for those of ASPS when compared to muscle. Of the seven other cases of primary ASPS, five documented MRI characteristics, where the tumours were all described as either hypo- to isointense on T1WI and markedly hyperintense on T2WI when compared to grey matter. Similarly, all lesions showed avid and heterogeneous enhancement on T1WI following contrast administration. Specific comment was made to the presence of flow voids within the tumour on T2WI in two cases; a characteristic noted in all 19 patients who underwent MRI in an oncological trial for treatment of extra-cranial ASPS [15]. Given this, primary ASPS may mimic meningioma on initial radiological investigation, with a differential diagnosis including hemangiopericytoma and medulloblastoma when encountered in the posterior fossa.

**Fig. 3:**
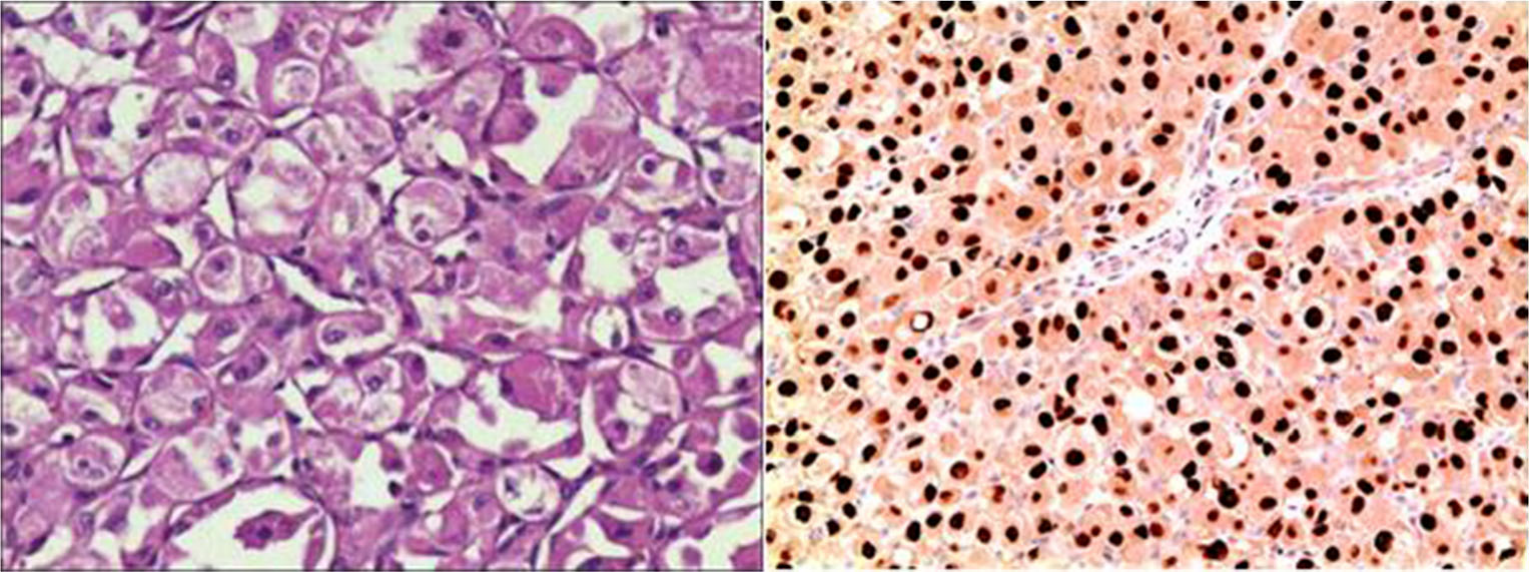
*Left*—Cells arranged in alveolar pattern with abundant granular cytoplasm (hematoxylin and eosin, ×100). *Right*—Positive nuclear uptake for TFE3 gene (TFE3, ×100)

Macroscopically, ASPS is typically a round or lobulated soft mass of a yellow-white color [16]. The histological diagnosis of ASPS is based upon its alveolar growth pattern and unique cytological features such as diastase-resistant periodic acid-Schiff (PAS-D) crystalline cytoplasmic inclusion bodies [4]. It is this characteristic histological appearance that has led to the nomenclature “alveolar soft part sarcoma” as it is comparable, although unrelated, to the alveoli of the lungs. Histological examination in our case did show PAS-D cytoplasmic positivity, but it is possible for ASPS tumours lacking these characteristic features to be mistaken for a wide differential of malignancies. This occurred in case 3 where a pre-operative histological diagnosis of meningioma was made following biopsy. The histological differential of ASPS includes the following: renal cell carcinoma (RCC); adrenocortical carcinoma (ACC); hepatocellular carcinoma (HCC); paediatric age RCC; malignant melanoma; paraganglioma; alveolar rhabdomyosarcoma; rhabdoid meningioma; perivascular epithelioid cell tumours (PEComas) and granular cell tumours. Given this broad differential, to confirm the diagnosis of ASPS, several specific immunohistochemical markers must be used to differentiate these separate malignancies. RCC can be excluded from ASPS as it shows a unique RCC marker protein. ACC will show immunoreactivity to synaptophysin, HCC will be positive for hepar-1 and rare paediatric RCCs are positive for CK. Similarly, alveolar rhabdomyosarcoma will show positivity for desmin, PEComas for smooth muscle actin and rhabdoid meningiomas will show eosinophilic inclusions strongly positive for EMA. Malignant melanoma, paraganglioma and granular cell tumours all stain positive for the S-100 protein (Fig. 3).

At times, the characteristic cytostructure that aids in the identification of ASPS can be absent. When this occurs, immunohistochemical testing for the nuclear transcription factor E3 (TFE3) fusion gene can be employed. Recently, an antibody directed against the C-terminus of the TFE3 fusion gene has proven a highly sensitive and specific marker for ASPS. Despite this, caution must be exercised as the TFE3 gene is also present, but in a different configuration, in paediatric RCC and also in granular cell tumours [2]. Therefore, to fully confirm the diagnosis of a primary intracranial ASPS, not only must the nuclear TFE3 fusion gene be detected, but other histological differentials must be excluded via specific molecular testing as well as the potential for any renal mass on imaging [15].

The cellular origin of ASPS remains an issue of debate and therefore makes investigation for the origin of primary disease difficult. Originally, it was thought that ASPS was a variant of granular cell tumour, but more recent immunohistochemical studies point towards a muscular origin [6]. Given the cellular origin of the ASPS remains ambiguous, there is no defined radiological protocol for the detection of primary disease. Excluding case 5 where details are not given, all patients had radiological imaging of the chest and abdomen, but the imaging modalities employed varied widely. CT of the chest, abdomen and pelvis was used in three cases to search for a primary; with CT of the chest alone in two further cases. Ultrasonography and bone marrow biopsy were used in four cases and whole-body PET in four cases. In case 2, a “bone scan” was performed but no further details of the exact imaging techniques used were given. FDG-PET scanning may be the most useful imaging modality in the search of an ASPS primary, with an estimated sensitivity of 93.7% in the detection of soft tissue sarcomas [14]. Therefore, to confirm a primary intracranial ASPS, an initial investigation for primary disease warrants detailed imaging of the chest to exclude pulmonary metastases and also whole-body FDG-PET in search of disease primary.

## Treatment

Chemotherapy, radiotherapy and surgical resection have been employed individually and in combination in the management of primary ASPS. Given the rarity of the disease, reports into the outcomes of these treatment modalities are limited to large case series and do not address ASPS effecting one specific anatomical region.

Surgery may be considered the first-line treatment in localized ASPS and may potentially increase long-term survival [22]. Prognostic factors that may guide the surgeon in counseling patients include: pre-operative Karnofsky Performance Score (KPS), tumour size, extent of surgical resection possible and patient age. A pre-surgical KPS of 70 and above has been reported as a good prognostic indicator associated with a higher median survival in those with metastatic sarcoma of the brain [3]. Primary tumour size is also thought to influence long-term survival [23]. In a series of 20 children and young adults with ASPS, progression-free survival was significantly reduced in those with a primary tumour greater than 5 cm in size [11]. Here, patients were treated with a combination of surgical resection, chemotherapy and radiotherapy dependent upon the extent of metastatic disease. Contrary to this, a more recent case series of 11 patients treated surgically concluded that neither tumour size, location nor interval between diagnosis and treatment effected outcome [7]. It is of note however in this series, all patients underwent total surgical excision and adjuvant radiotherapy with the authors conceding their number of cases being too small to perform meaningful analysis.

A favorable outcome from complete microscopic resection of localized ASPS has been reported from several series. A study by the Italian Soft-Tissue Sarcoma Cooperative Group found that 100% of patients who underwent total surgical resection of localized ASPS showed no evidence of disease at follow-up ranging from 11 to 243 months, often with surgery being the only treatment modality employed [5]. Similar outcomes have been confirmed in the paediatric and young adult population following total surgical resection of primary disease [11, 18]. Increased age at diagnosis has been shown to have an association with worse prognosis, but this correlation may be due to younger patients presenting with more localized disease when compared to those aged 17 years and above [11, 12]. Disease-free and overall survival rates in those with localized ASPS have been reported at 71 and 88% at 5 years, respectively [20].

In our review of primary intracranial ASPS, total surgical resection was achieved in six of eight cases. Total surgical resection was deemed unsafe in two cases due to difficult to control large-volume haemorrhage. Three out of the seven cases were detailed experienced localized disease recurrence, with two of these patients having undergone total surgical resection and the other subtotal resection. In two instances, patients received adjuvant chemo- and radiotherapy and in the other radiotherapy alone. Of the four patients who remain disease-free, two underwent total surgical resection alone, one underwent total surgical resection plus adjuvant chemotherapy and one total surgical resection plus radiotherapy.

It is of note that in the three instances of disease recurrence, the follow-up time is significantly longer than in those who remain disease-free. Also, contrary to the association between younger age at presentation and favorable prognosis reported from extra-cranial primary ASPS, the only three patients under 17 years of age in this review were the ones to demonstrate disease recurrence.

ASPS appears to be a chemotherapy-insensitive pathology. Eleven patients with both localized and metastatic ASPS who received chemotherapy failed to show any response to treatment. Chemotherapeutic agents used were varied including antimetabolites, alkylating agents, mitotic inhibitors, anthracyclines, antimicrobial agents, antineoplastics, antifolates, COX-2 inhibitors, interleukin-2, interferon-alpha and TNP-470 [11]. The largest report of results from chemotherapeutic treatment in ASPS showed only 3% of patients responded to doxorubicin-based therapy. It is of note that total number of patients was low at 29 but did include patients with both localized and metastatic ASPS. The majority of patients treated with chemotherapy developed disease progression, and no partial or minor responses were seen [20].

The role of radiotherapy in the treatment of ASPS remains unknown. The few case series documenting the response to radiotherapy are unable to demonstrate any clinical benefit due to small study numbers [11, 20]. Radiotherapy has been employed in an adjuvant and palliative fashion. In those patients where adjuvant radiotherapy was used, the decision to do so was based upon the perceived high risk of recurrence for their tumours. Clinico-pathological factors used for this assessment were microscopically positive surgical margins, anatomic location, presentation with a recurrent primary site and tumour size. A study of 18 patients focusing on the role of radiotherapy in ASPS concluded that it may be of use in the prolonged local control of disease after limited surgical resection and also provide meaningful palliation in metastatic ASPS [21].

As the long-term prognosis for localized ASPS is favorable, patients will require extended periods of follow-up with appropriate cranial imaging. Cases 3 and 4 highlight the need for extended follow-up as disease recurrence can occur in a delayed fashion. Also, extended follow-up is recommended to ensure that no initially occult primary disease focus renders itself detectable. Furthermore, even in patients with widespread metastatic disease, there exists an unexplained subset of long-term survivors beyond 5 years from initial treatment. These patients highlight both the potential indolent nature of even metastatic ASPS and our incomplete understanding of its natural history. In time, molecular analysis of these patient subsets may provide an explanation for this variation in survival.

## Clinical implications

Based upon the clinical features of the reported cases of primary intracranial ASPS in conjunction with literature to date, there are several implications for consideration in the management of this disease. First, our case gives further evidence that ASPS may arise as a primary intracranial malignancy within the posterior fossa. Second, to confidently demonstrate a primary intracranial ASPS, whole-body FDG-PET scanning must be performed to exclude a soft tissue primary as well as CT imaging of the thorax to rule out pulmonary involvement. If unable to test for the TFE3 fusion gene, then imaging of the kidneys should also be performed to exclude a renal mass. Third, primary intracranial ASPS should be borne in the mind of the neurosurgeon when confronted with what radiologically appears to be an atypical meningioma, haemangiopericytoma or medullablastoma in the younger patient. Fourth, total surgical resection of primary disease is the main treatment option as neither chemotherapy nor radiotherapy has proven benefit in curative management in extra-cranial disease. Furthermore, where the benefit of a diagnostic biopsy is available prior to resection, pre-operative preparation for large-volume haemorrhage is advised. Fifth, contrary to the trend in extra-cranial primary ASPS, younger patients with primary intracranial ASPS may have a worse prognosis.

## Conclusions

At this moment, we know neither the natural history nor the cellular origin of this primary intracranial malignancy. Our case makes part of this review which is the most comprehensive to date of this disease. Still, we cannot strongly conclude what may be the best treatment options for primary intracranial ASPS from these few cases, despite significant literature documenting experience with extra-cranial disease. We therefore encourage other clinicians to report their encounters with this new primary intracranial malignancy in the endeavor to create a greater understanding of this disease and its best management.
